# Development of speech intelligibility in geriatric individuals with hearing loss

**DOI:** 10.3906/sag-2002-200

**Published:** 2021-12-16

**Authors:** Bünyamin ÇILDIR, Meral Didem TÜRKYILMAZ

**Affiliations:** 1 Department of Language and Speech Therapy, Faculty of Health Sciences, Ankara Yıldırım Beyazıt University, Ankara, Turkey; 2 Department of Audiology, Faculty of Health Sciences, Hacettepe University, Ankara, Turkey

**Keywords:** Bilateral/unilateral hearing aids, bicros, adaptive Turkish matrix test, speech in noise, speech intelligibility

## Abstract

**Background/aim:**

In our study, we aimed to evaluate the hearing aid benefit and speech intelligibility with hearing aids using objective and subjective measurements, according to the type of hearing loss in elderly individuals who used different types of hearing aids.

**Materials and methods:**

The objective and subjective findings from a total of 47 elderly individuals between the ages of 60 and 84, who used regular hearing aids for at least six months, and who were diagnosed with different types and degrees of hearing loss were evaluated by scanning them retrospectively.

**Results:**

In our study, the Adaptive Turkish matrix sentence test (ATMST) was carried out with binaural headphones, and a statistically significant difference was observed between the ATMST scores of individuals with symmetrical hearing loss. A significant difference was found between the ATMST score averages for individuals with symmetrical hearing loss (S^0^N^90^ and S^0^N^270^) and asymmetric hearing loss (S^0^N^0^ and S^0^N^270^) in the free area. A significant difference was found between abbreviated profile of hearing aid benefit satisfaction questionnaires before and after hearing aid use in all groups.

**Conclusion:**

The Turkish matrix sentence test (TMST) in noise can be used routinely in clinics in order to evaluate the possible hearing loss from the daily environment and the hearing aid effectiveness.

## 1. Introduction

Hearing loss, which is one of the most common neurological diseases and emerges with old age [1], is a health problem which has affected 466 million people worldwide (according to the data of the World Health Organization) and it can affect 630 million people until 2030 and 900 million people (one-in-10 people) until 2050^1^. It is thought that age-related hearing loss stems from the damage to cells or structures that are responsible for sound conduction and coding, due to noise trauma, microvascular trauma, and many other reasons [2]. Although Presbycusis (generally progressive bilateral symmetrical sensorial hearing loss) [3] is the most commonly seen hearing loss in the population of people older than 65, the hearing loss with different types and extent is also met [4]. When there is no clinical or surgical treatment to be applied for this type of hearing loss, hearing aid application is generally used as a rehabilitation option. The improvement of speech intelligibility (especially fast speech and background noise cases) is the most important objective of the hearing aid application [5]. It was also stated that the bilateral device application, being different than a single-sided hearing aid, led to positive changes in speech intelligibility skills due to binaural squelch, binaural redundancy, and the improvement of the signal-noise ratio [5]. There are many test batteries (HINT, matrix sentence test in noise/in quiet, SPIN) used for evaluating hearing aid effectiveness [6, 7]. These are different not only in terms of the speech materials (logatomes, monosyllables, numbers, and meaningful or meaningless sentences) but also in terms of the application models (presentation in quiet or in noise, fixed or adaptive levels of speech and/or noise level, and type of noise). The Matrix sentence test [8–10], which has been translated into many languages around the world, is one of the tests developed for distinguishing sentences in noise like HINT and SPIN which is commonly used in medical research and clinical environments. This test can determine the communication status in daily situations together with the sentence in noise test and speech intelligibility test. Furthermore, it gives additional information about real hearing disorders in the individuals using hearing aids/cochlear implants [11–13]. TMST was first devised by Zokoll et al. in order to evaluate and study the speech intelligibilities of individuals who have normal hearing and who have hearing loss [11]. TMST was developed to determine speech intelligibility in different hearing cases (different background noise types, speaking from different azimuths, and not coming from the noise) [14].

In accordance with the aim of this study, the speech intelligibilities of geriatric individuals with hearing loss were determined through sentence tests by performing TMST in noise with constant talk simulation. Then, it was also aimed to decide the appropriate amplification for every patient and to determine how much they benefit from the use of hearing aids and the superiority of the single-sided/bilateral hearing aids.

## 2. Materials and methods

### 2.1 Participants

In our study, the patients (47 patients with hearing loss), who applied to the Audiology Unit of Hacettepe University Adult Hospital between 2015 and 2018 due to hearing loss and were given hearing aids and were followed for 6 months at least, were scanned retrospectively. Among those individuals, 44 individuals with hearing loss (29 individuals with symmetrical sensorineural hearing loss (16 men and 13 women), 8 individuals with asymmetrical sensorineural hearing loss (4 men and 4 women), and 7 single-sided sensorineural hearing loss (3 men 4 women)), who came to follow-ups regularly and who had other tests done besides routine ones, were included in the study. Individuals were divided into three groups (symmetric, asymmetric, and unilateral hearing loss) according to hearing loss type. Patients’ pure-tone air conduction thresholds (between 125 and 8kHz octave frequency) were tested via a GSI audiometry device. The air conduction pure tone averages (PTA) of individuals with asymmetrical hearing loss were determined as such; for right ear 40.31 ± 29.05; for the left ear, 71.87 ± 21.82. The PTA of individuals with left ear single-sided sensorineural hearing loss was determined as such: for right ear 33.75 ± 8.75, for left ear 92.50 ± 10.68. The PTA of individuals with right ear single-sided sensorineural hearing loss was determined as such: for the right ear, 10 ± 1.44; for the left ear, 120. The PTA of individuals with symmetrical hearing loss was determined as such: for the left ear, 46.29 ± 11.03: for the right ear, 46.89 ± 9.99. Age averages of all the individuals were determined to be 68.02 ± 5.45 (between the ages of 60 and 84). MOBID tests were done on all of the patients, who were given hearing aid and came to their follow-up appointments, and only one of the patients’ test scores was less than 21 in this test. However, the patient who got less than 21 was not included in the study as he did not come to the follow-up. Demographic information about the individuals is shown in Table 1. The Helsinki Declaration was filled for retrospective scanning of the patients, and approval was given from the ethical committee for retrospective scanning of the data. In the tests, which were done apart from the routine tests carried out in clinic, the patients were given preliminary information and those tests were done on different days from the routine tests by the same expert.

### 2.2. The self-report questionnaire

The EQ-5D-3L scale for general life quality and the satisfaction questionnaire for hearing aid (abbreviated profile of hearing aid benefit (APHAP)), which were carried out with the patients’ permission, were scanned retrospectively.

### 2.3. The abbreviated profile of hearing aid benefit (APHAB)

The Turkish satisfaction questionnaire for hearing aids given to the patients for the assessment was examined retrospectively. APHAB, which evaluates different situations with and without hearing aids, consists of 4 subgroups (ease of communication, communication in rooms with echo and/or reverberation, communication in the presence of high pitch sounds in the background, and lack of acceptation of unexpected sound coming from the environment) and 24 items. Those items help patients and clinicians assess performance with and without hearing aids. In the questionnaire interpretation, the averages of the questions asked in each of the subgroups (averages of 4 subgroups) show the APHAB score [15].

### 2.4. EQ-5D scale for general life quality

The validity and reliability studies of the EQ-5D-3L scale for general life quality, which was developed to assess the general life quality of many disease groups, were carried out by H. Kahyaoğlu Süt (2009) [18]. The scale consists of two parts. In the first part, the EQ-5D index scale consists of five aspects: mobility, self-care, usual activities, pain/discomfort, and anxiety/depression. In the second part, there is a scale entitled EQ-5D VAS used to assess the current health conditions of the individuals rated between 0 and 100. For each question, the responses are as follows: there is no problem, there are some problems, and there is a big problem. In EQ-5D index score calculation, the coefficients determined by Dolan et al. [16] were used [17].

### 2.5. Speech materials

The TMCT, developed by Zokoll et al. (2015), was applied to the patients in order to evaluate their skills of distinguishing speech in noise and speech intelligibility [11]. TMCT was created according to an open-ended presentation model. In this test, the individuals are asked to repeat all of the words in a given sentence. Each of the correctly repeated words is recorded by the test operator using a touch screen. According to the number of correctly understood words in a sentence, the level of speech changes adaptively depending on the increase in the score of correctly repeated words. The procedure starts with a 65 dB SPL constant noise level and a 0 dB SPL signal-noise ratio. When 50% of the given words are understood correctly, the presentation level is determined for the new sentence. Speech Recognition Threshold (SRT) evaluation is specified via the maximum possibility method [18]. The noise used in the Matrix sentence test is the noise of speech simulation with the same length average spectrum [19]. The noise stimulus given during the test starts 500 ms before each of the sentences and ends 500ms after giving the stimulus. The level of the signal-noise ratio is determined separately for each ear. While the level of noise remains constant in 65 dB SPL, the signal adaptation procedure (when PTA > 55, the level of noise remains constant and the signal level is given in 80 dB SPL) starts at the 65 dB SPL signal level (0 dB SNR). The measurements were completed under the same conditions independently from the threshold of all the individuals [11, 20].

The adaptive Turkish matrix sentence test (ATMST) was performed on all of the individuals in a sound-free cabin using Sennheiser HDA200 headphones (without hearing aids and using both ears at the same time (bilaterally)). In the same session, the individual was asked to sit between the two speakers in the cabin (distance to a speaker is 1 m) and free field tests were performed. One of the speakers was placed just in front of the patient and the other speaker was placed on the patient’s right. The patient was tested adaptively, with a randomly selected list of 20 sentences, as follows: The stimuli always came from in front of the patient; first the noise and signal came from just in front of the patient (S^0^N^0^); then when the signal came from the front, the noise came from the right side of the patient (S^0^N^90^); and lastly, the patient turned to the speaker on his or her right side and the signal came from the front and noise came from the left side of the patient (S^0^N^270^). The ATMST in noise –with and without a device- was performed and questionnaires were given to all of the patients 1 month, 6 months, and 12 months after the application of hearing aids. However, since among the patients who were included in the study, only 2 of them came to annual control, the results for 6 months were given and annual results were discussed. The TMST was tested through Sennheiser HDA200 headphones by using an otometrics audiometry device (AURICAL Aud, Otometrics, Taastrup, Denmark) connected to a PC. Calibration of the device was carried out regularly and monthly in accordance with American Speech Hearing Association (ASHA) standards [21]. Materials belonging to TMCT were given with computer-based Oldenburg matrix test application software. Free field tests were given via free field speakers according to international standards (ISO 389-8), and measurement standards were calibrated according to the standards specified by the producer (HörTech GmbH, Oldenburg, Germany).

### 2.6. Statistical analysis

The analyses of the data, which were scanned retrospectively, were carried out using the IBM SPSS version 23.0 computer program. The nonparametric Wilcoxon signed rank test was used in the repeated dependent data analysis. The Mann-Whitney U test was also employed in the statistical analyses between the 2 groups. For all analyses, effects were significant with p < 0.05.

## 3. Results

**Figure 1:**
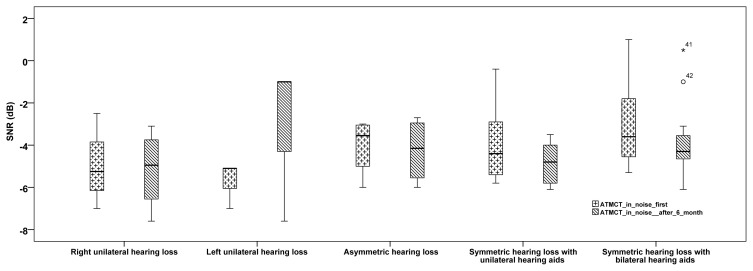
Finding of ATMST in noise test with binaural headphone before and after using hearing aids for all individuals with hearing loss. ATMST_in noise first shows Turkish matrix test in noise results before using hearing aids, ATMST_in noise after 6 months shows results average of 6 months using hearing aids.

The ATMST in noise tests, which were carried out with binaural headphones belonging to all individuals in the study, are presented in Figure 1. Before the individuals used the hearing aids, and at least 6 months after they used them, a statistically significant difference was not found between the SNR average values of the ATMST in noise test with headphones (with a noise level of 65 dB SPL) of the individuals with right and left-ear single-sided hearing loss and asymmetrical hearing loss (p > 0.05). However, compared to their previous situations, a minimal increase was observed in the SNR ratios of the individuals with asymmetrical and single-sided hearing loss. A statistically significant difference was found between the SNR average values of the ATMST in noise test of the individuals with symmetrical hearing loss (p = 0.035). The statistical results of the adaptive Turkish matrix sentence test of the individuals with symmetrical hearing loss and with hearing aids are presented in Table 2.

**Figure 2:**
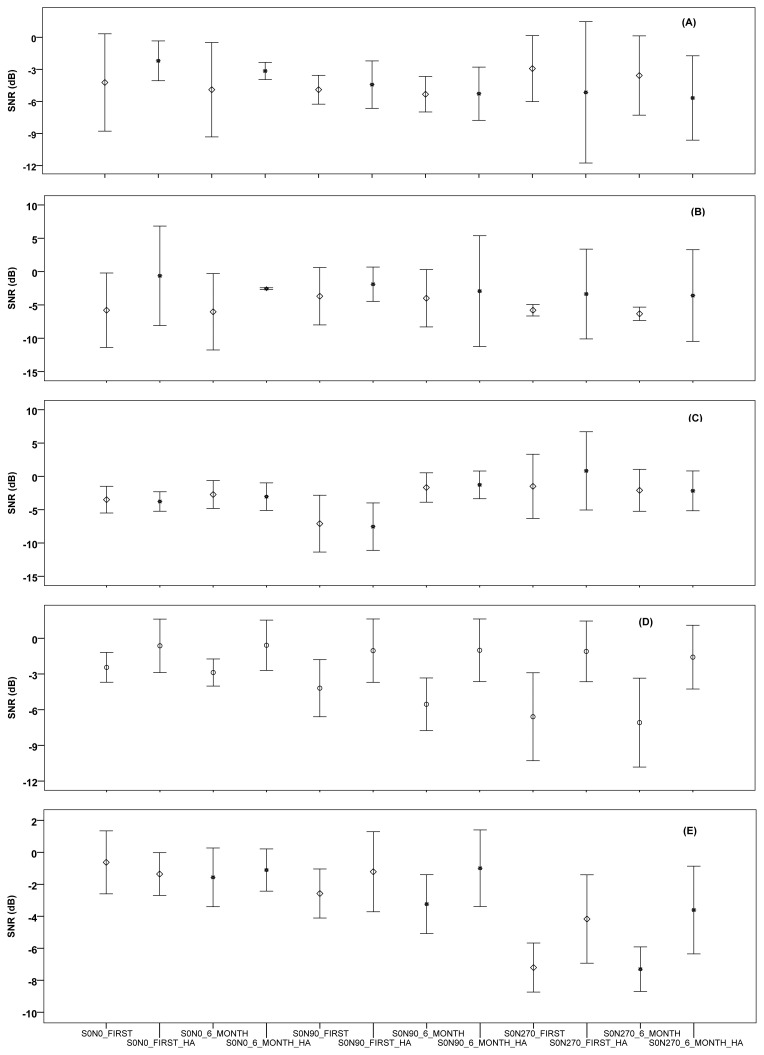
Findings of free-field TMST in noise S^0^N^0^/S^0^N^90^/S^0^N^270^ SNR of unilateral, asymmetric and bilateral hearing-loss individuals without and after hearing aid use; A: right unilateral hearing loss; B: left unilateral hearing loss; C: asymmetric hearing loss; D: Symmetric hearing loss with unilateral hearing aid; E: Symmetric hearing loss with bilateral hearing aid.

The results of the free field ATMST in noise SNR averages of the individuals with bilateral and single-sided hearing loss in the presence of noise from different directions are shown in Figure 2. The free field ATMST in noise SNR averages of S^0^N^0^, S^0^N^90^, and S^0^N^270^ were carried out before the hearing aids were used, after the hearing aids were used, and without the hearing aids in constant noise. A statistically significant difference was not found in the individuals with right and left-ear single-sided hearing loss, asymmetrical loss using single-sided (right ear) hearing aids, and with symmetrical hearing loss (p > 0.05).

However, compared to score averages taken without hearing aids, an increase was observed in all individuals’ score averages recorded 6 months after using the hearing aids. Furthermore, in the free field ATMST performed before using the hearing aids and 6 months after using the hearing aids, a statistically significant difference was found between the score averages of the individuals with symmetrical hearing loss using single-sided (left ear) hearing aids (p > 0.05). A statistically significant difference was found in the S^0^N^0^ (p = 0.032) and S^0^N^270^ (p = 0.029) averages of the individuals with asymmetrical loss and with single-sided hearing aids in free field ATMST with hearing aids (their score was much higher when the noise came from the side with the hearing aid) (as shown in Table 2). S^0^N^0^, S^0^N^90^, and S^0^N^270^ free field tests were carried out before and after the hearing aids were used. In the case of with and without constant noise, a statistically significant difference was not found between the SNR rates of S^0^N^0^, S^0^N^90^, and S^0^N^270^ free field tests of the individuals with right and left single-sided hearing loss (p > 0.05). In the patients with symmetrical hearing loss using bilateral hearing aids, a significant difference was detected between the SNR average values, which were obtained in the case of without hearing aids and with hearing aids S0N90 (p = 0.030) and S^0^N^270^ (p = 0.017) (as shown in Table 2). Lastly, a significant difference was not found between the S^0^N^0^, S^0^N^90^, S^0^N^270^ results of the individuals with symmetrical hearing loss using single-sided hearing aids and the results of individuals using bilateral hearing aids (p > 0.05).

A statistically significant difference was not found in the general health conditions of the patients in the EQ-5D-3L scale for general life quality (mobility, self-care, usual activities, pain/discomfort, and anxiety/depression) (p > 0.05).

A statistically significant difference was found between the average values in the abbreviated profile of hearing aid benefit questionnaire (APHAB) in terms of the situation before and after the use of hearing aids (p = 0.026). It was also observed that after the use of hearing aids, the communicative skills of the individuals increased (p = 0.001), echoing decreased (p = 0.078), communication in the case of background sounds increased (p = 0.001), and lack of acceptation of unexpected sound coming from the environment decreased (p= 0.015). The mean values and standard deviations of the APHAB subscale of individuals with and without hearing aids are presented in Table 3. It was also determined that the hearing aid satisfaction questionnaire scores of the individuals using single-sided hearing aids were better than those of individuals in the bilateral group, but a statistically significant difference was not found.

## 4. Discussion

In this study, the aim was to determine the speech intelligibility of geriatric individuals with hearing loss to whom ATMST was performed in constant speech simulation noise. Then, another goal was to be able to decide on the appropriate amplification choice for each of the patients (unilateral or bilateral hearing aids) and to determine how much they benefitted from hearing aid usage in the rehabilitation process. It was thought, in accordance with the findings obtained in our study, that the improvements in the speech intelligibility in changing noisy situations may occur not only in the individuals with bilateral hearing loss and with hearing aids, but also in the individuals with single-sided hearing loss and with BICROS/CROS hearing aids. Therefore, it was believed that giving appropriate amplification to the patients with single-sided hearing loss may change their speech intelligibility in noise positively. In our study, an improvement (but not a statistically significant one) was observed in the speech intelligibility of individuals with single-sided hearing loss when they had BICROS hearing aid on their ear and the noise was given to functional ear. It was thought that the situation could stem from the adaptation of patients with single-sided hearing loss [22,23] to pinna effectiveness depending on the sound clues coming vertically for distinguishing the sounds coming horizontally from different directions. It was indicated that the individuals with single-sided severe and very severe sensorineural hearing loss had difficulty in determining the localization of the sounds coming horizontally from the direction of the bad ear [24] and in understanding speech, especially in noisy environments although their one ear was functional [25–29]. It was also articulated that the disorder stemmed from the shadow effect of the head and binaural hearing loss [24]. In our study, it was found that in the individuals using BICROS hearing aids, there was an increase (in the measurements without hearing aid) in their speech intelligibility compared to their earlier situation (according to the SNR average of ATMST in noise coming from the bad air in different azimuths). Also, that situation showed the effectiveness of hearing aids in the individuals with single-sided hearing loss. It was also thought that being unable to find significant differences between the averages obtained from the individuals could be caused by the rareness of people we could follow due to the limited number of people with single-sided hearing loss and with BICROS/CROS hearing aids in our country. As previously stated, in our study, a significant difference was found between the results of the individuals with single-sided/bilateral hearing loss and with hearing aids obtained from S^0^N^90^ and S^0^N^270^ free field ATMST in noise. This result showed that when both the signal and the noise are given from the front of the patient, it becomes more difficult for patients to distinguish speech compared to other cases. In addition, it was also believed that such a result could stem from the fact that the hearing losses of the individuals were different depending on the individuals’ ages or that there was a lack between the estimated benefit of the hearing aid (NAL-NL1) and the hearing loss in the geriatric individuals. Observation of the difference between SNR rates obtained in the skill of speech distinguishing when the noise stimuli came from 90° and 270° azimuths of the patients led to the thought that, especially in geriatric individuals determining the hearing aid strategy, in which speech stimuli could be used most effectively, depending on the changing noise direction would increase the benefit the patients could have from the device. In the HINT test, which was performed on the individuals who spoke Turkish and had a normal hearing threshold, the following results were found: when the noise was in front of the individual (S^0^N^0^), −3.2 ± 1.1 dB SNR; when the noise was on the right of the individual, −11.5 ± 1.3 dB SNR; and when the noise was on the left of the individual, −11.8 ± 1.2 dB SNR (31). In our study, when the signal and noise were given just from the front of the patients, more difficulty in understanding speech was observed compared to other cases, and this result showed the similarity between this study and the studies carried out by [6] and [30].

Not observing a significant difference between free field ATMST in noise (S^0^N^0^,S^0^N^90^, and S^0^N^270^) SNR rates of the individuals with single-sided/bilateral hearing loss (but the scores of the individuals using bilateral hearing aids increased more than those using single-sided hearing aid) was found compatible with the other studies [31,32]. It is required to assess the perceptive development of the speech intelligibility in the individuals, who have just used hearing aids, depending on their individualistic characteristics (personality, motivation, and expectations) [33,34]. The general life quality scores of the individuals who were included in the study did not change after they used hearing aids. However, it was found that the difference in noise scores of the individuals, who had high-quality daily life was better than other individuals. In all of the hearing aid satisfaction questionnaires, significant differences were observed in each of the subtests. A significant difference could not be found between the APHAB questionnaire scores of the individuals using single-sided hearing aids and those using bilateral hearing aids. However, an increase was seen in the score in favor of the individuals using bilateral hearing aids only in one of the subtests of the questionnaire: speech intelligibility when there is background noise. It was thought that since bilateral hearing aids are effective on both ears when noises come from all directions, they can be beneficial under complex noise plans (like in restaurants) in terms of increasing the speech intelligibility of individuals compared to a single-sided hearing aid. The reason that a significant difference could be found between the individuals using bilateral hearing aids and those using single-sided hearing aids (even the majority of the patients wanted to use a single-sided hearing aid) could be the fact that, as Dillon et al. stated, the hearing aid, which is programmed differently, can change interaural time and loudness clues and the use of bilateral hearing aids cause negative effects on binaural clues [35, 36].

## 5. Conclusions

Our study is important for this field because there have been no studies carried out with hearing aids (in different types) using the matrix sentence test. In our study, an increase in the speech intelligibility of the individuals using BICROS hearing aids was observed compared to their earlier situations without the hearing aids. Giving hearing aids to geriatric individuals, whose one ear is functional and who have single-sided hearing loss, not only provides an increase in the speech intelligibility in noise but also increases their life quality. It is believed that the inclusion of Turkish matrix sentence test, which is different from other routine audiological tests, in routine clinic usage for determining the amplification can give useful results for evaluating and following the patients with different types of hearing loss. The findings obtained were compatible with the study of Prates et al., in which the authors evaluated speech intelligibility and SNR rates of patients with hearing loss who used hearing aids for three months [37].

**Highlights**
- The Turkish matrix sentence test showed that there was an improvement in the speech intelligibility of elderly individuals with hearing loss after hearing aid use.
- The use of bilateral hearing aids provides more benefits than a unilateral hearing aid.
- Objective and subjective evaluation is done together to evaluate the benefit of hearing aids in elderly individuals.

## Figures and Tables

**Table 1:** Demographic data for each patient.

	N	Men/women	Age (years)	Legent of HA use (month)	A daily time of HA user (h)	Directional of HA
**Right unilateral HL**	4	1/3	67.5(2.08)	7.7 (1.25)	11(2.58)	Bicros
**Left unilateral HL**	3	2/1	71.66(1.52)	12 (2)	9 (2.64)	Bicros
**Asymmetric HL**	8	4/4	67.5(2.61)	8.7(2.5)	8.12(1.1)	4 right/4 left
**Symmetric HL with unilateral HA**	13	9/4	66.15(4.09)	8 (2.51)	9.07(2.59)	8 right/5 left
**Symmetric HL with Bilateral HA**	16	7/9	69.62(7.62)	7.68(1.49)	8.85(1.59)	Bilateral

HL: Hearing loss, HA: Hearing aids

**Table 2:** Statistical results of the adaptive Turkish matrix sentence test of the individuals with hearing aids.

	Results after using the first hearing aids		Results 6 months after using the hearing aids		P-value
	Mean ± SD	Min/max	Mean ± SD	Min/max	
**Symmetric hearing loss**					
**ATMST in noise test with headphone**	−3.33 ± 2.06	−5.8 / 1	−4.35 ± 1.46	−6.1/0.5	**0.035** *
**Symmetric hearing loss with unilateral hearing aids**					
**ATMST in noise test with free field (S** **^0^** ** N** **^0^** **)**	−0.63 ± 2.1	−5.8/7.3	−2.87 ± 1.88	−5/2.1	**0.032** *
**ATMST in noise test with free field (S** **^0^** ** N** **^270^** **)**	−2.44 ± 2.07	−5/2.4	−4.11 ± 3.97	−8.5/4.7	**0.029** *
**Symmetric hearing loss with bilateral hearing aids**					
**ATMST in noise test with free field (S** **^0^** **N** **^90^** **)**	−0.62 ± 3.69	−4.8/6.9	−1.56 ± 3.44	−5.9/5.1	**0.030** *
**ATMST in noise test with free field (S** **^0^** **N** **^270^** **)**	−2.51 ± 2.2	−5.3/1	−3.98 ± 1.72	−6.1/0.5	**0.017** *

ATMST: Adaptive Turkish matrix sentence test; SD: Standard Deviation; Min: Minimum; Max: Maximum;

* p < 0.05

**Table 3:** Mean values and standard deviations of the APHAB subscale of the participants with and without hearing aids.

	Mean ± SD with hearing aids	Mean ± SD without hearing aids	P-value
**Overall averages**	2.38 ± 0.57	4.12 ± 1.02	**0.026** *
**Ease of communication**	2.84 ± 1.1	5.40 ± 1.42	**0.001** *
**Background noise**	2.19 ± 0.9	5.21 ± 1.14	**0.001** *
**Reverberation or echo**	3.01 ± 1.14	3.57 ± 1.03	0.078
**Aversiveness**	5.42 ± 1.04	4.10 ± 1.57	**0.015** *

SD: Standard deviation;

* p < 0.05

## Footnotes

1 WHO (2019). Deafness and hearing loss (online). Website: https://www.who.int/news-room/fact-sheets/detail/deafness-and-hearing-loss [Accessed on 06 November 2019].
